# Hyperbaric oxygen therapy and N-acetylcysteine: a redox-dependent interaction

**DOI:** 10.3389/fmed.2026.1829074

**Published:** 2026-04-13

**Authors:** Sanjin Kovacevic, Aleksandra Nenadovic, Rada Jeremic, Milan Ivanov, Predrag Brkic, Nevena Mihailovic-Stanojevic, Jelena Nesovic Ostojic

**Affiliations:** 1 Department of Pathological Physiology, Faculty of Medicine, University of Belgrade, Belgrade, Serbia; 2 Department of Medical Physiology, Faculty of Medicine, University of Belgrade, Belgrade, Serbia; 3 Department of Cardiovascular Physiology, Institute for Medical Research, National Institute of Republic of Serbia, University of Belgrade, Belgrade, Serbia

**Keywords:** antioxidants, hyperbaric oxygenation, N-acetylcysteine, oxidative stress, redox homeostasis

## Abstract

Hyperbaric oxygen therapy (HBOT) is widely used in clinical medicine and exerts its therapeutic effects primarily by modulating cellular redox signaling. Acute exposure to hyperbaric hyperoxia induces a transient increase in reactive oxygen species (ROS), which can initiate adaptive antioxidant responses and activate cytoprotective pathways. However, excessive ROS generation may also contribute to oxidative injury, depending on the treatment protocol and underlying pathological state. N-acetylcysteine (NAC), a precursor of glutathione and a widely used antioxidant, has therefore been investigated as a potential adjunct therapy to modulate HBOT-induced oxidative responses. This mini-review summarizes current evidence regarding the interaction between HBOT and NAC in experimental and clinical studies. The available studies indicate that the combined effects of HBOT and NAC are context-dependent. In conditions characterized by severe oxidative stress, NAC may enhance the therapeutic effects of HBOT by reducing pathological ROS accumulation and improving antioxidant capacity. In contrast, in models where ROS act as signaling molecules that trigger adaptive or regenerative pathways, antioxidant intervention may attenuate or abolish the beneficial effects of HBOT. Evidence also suggests that treatment timing, dosage, and baseline oxidative status may modify the outcomes of combined therapy. Overall, the interaction between HBOT and NAC reflects a delicate balance between pathological oxidative stress and redox-dependent signaling mechanisms. A better understanding of these dynamics may help optimize therapeutic strategies involving hyperbaric oxygen and antioxidant modulation in clinical practice.

## 1 Introduction

Hyperbaric oxygen therapy (HBOT) is a treatment that involves breathing pure oxygen under increased atmospheric pressure. According to the Undersea and Hyperbaric Medical Society (UHMS), HBOT consists of breathing nearly pure oxygen while being exposed to pressures of at least 1.4 absolute atmospheres (ATA) in a hyperbaric chamber. Currently, 15 indications for HBOT have been approved. However, all indications require patients to breathe nearly 100% oxygen in a pressurized chamber at a minimum of 2 ATA (1, 2).

Despite decades of intensive research, the full range of HBOT beneficial effects remains incompletely understood, even though numerous mechanisms have been proposed. HBOT can directly affect gene expression, signal transduction, mitochondrial function, and cell apoptosis, as well as induce the expression of antioxidant enzymes (3). Additionally, HBOT exhibits significant immunomodulatory and anti-inflammatory properties (2). Although HBOT can increase the production of reactive oxygen species (ROS), it may simultaneously initiate adaptive antioxidant responses that could be beneficial. Importantly, the magnitude of this adaptation is strongly influenced by the treatment protocol, particularly session duration, oxygen pressure, and treatment frequency (4–6).

**FIGURE 1:**
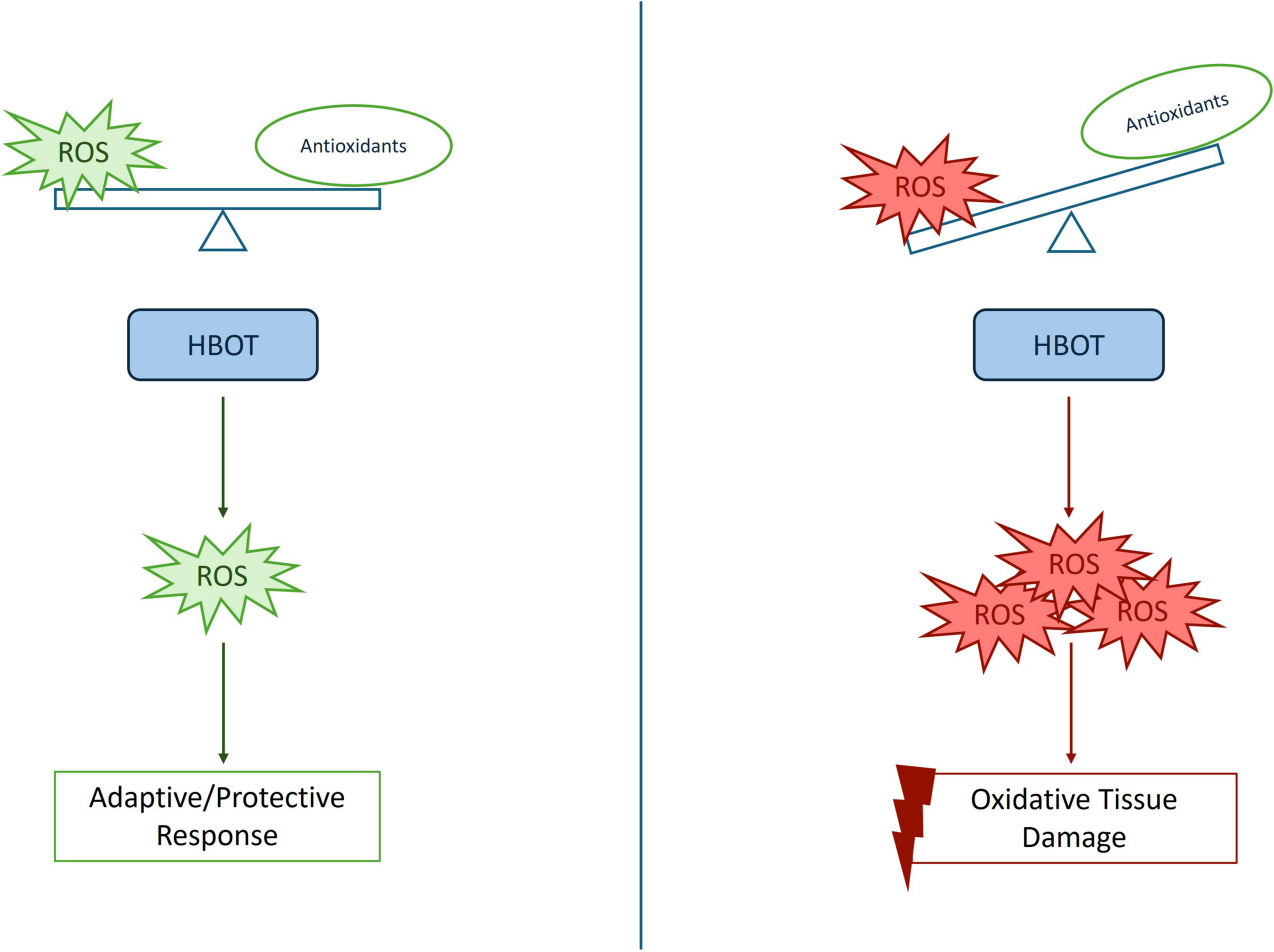
Hyperbaric oxygen therapy (HBOT)-induced reactive oxygen species (ROS): physiological signaling versus oxidative stress. HBOT increases tissue oxygen availability, leading to enhanced ROS production. At controlled levels (left), ROS act as a signaling molecules that activate adaptive pathways. In contrast, excessive ROS production (right), which may depend on HBOT protocol or underlying pathological conditions, leads to oxidative tissue injury.

Overall, evidence from *in vitro*, animal, and clinical studies suggests that HBOT primarily acts as a redox-modulating stimulus (Figure 1). Acute HBO exposure induces a transient increase in ROS generation, resulting in an initial pro-oxidative effect (7–11). However, this temporary ROS burst activates redox-sensitive transcriptional pathways, including nuclear factor erythroid 2-related factor 2 (Nrf-2), heme oxygenase-1 (HO-1), and hypoxia-inducible factor-1 alpha (HIF-1α), which subsequently promote upregulation of endogenous antioxidant and cytoprotective systems (7–12). This transient, controlled increase in ROS can further suppress nuclear factor kappa-light-chain-enhancer of activated B (NF-κB) cell signaling (13).

Vezzoli et al. (14) described the concept of oxy-inflammation in the context of underwater activities, where divers are exposed to alternating hypoxic and hyperoxic conditions under hyperbaric stress. This physiological setting represents a naturally occurring hormetic model, in which fluctuations in oxygen availability, ambient pressure, and environmental stressors collectively influence redox regulation and inflammatory signaling pathways (15). Extending these observations beyond traditional diving physiology, although seemingly paradoxical, transient hyperoxia followed by normoxia is perceived as relative hypoxia and can trigger protective, hypoxia-like responses without true hypoxic injury, consistent with previously described mechanisms (16, 17).

While intermittent HBOT often leads to a delayed improvement in redox balance, suggesting adaptive regulation rather than cumulative damage, this response is neither universal nor guaranteed (2, 18). Despite its broad applications and benefits, HBOT must be administered carefully, as it can cause oxidative stress and cell damage through excessive ROS production (2). It should also be recognized that in patients with a high baseline oxidative burden or severe, persistent oxidative stress, adaptive mechanisms may be insufficient, leading to prolonged redox imbalance (18, 19). One potential strategy involves combining antioxidants. Given the prominent role of redox modulation in hyperbaric oxygen biology, this mini-review specifically examines N-acetylcysteine (NAC), the most extensively studied antioxidant in this context, focusing on evidence from experimental *in vitro*, animal, and human models.

## 2 Antioxidants

Antioxidants play a crucial role in controlling free radicals and mitigating oxidative stress, a process linked to numerous diseases. By reducing oxidative damage, antioxidants preserve cellular integrity and support physiological function (20). Elevated dietary intake and plasma levels of antioxidants have been shown to have a significant inverse association with the risk of all-cause mortality (21). The body maintains redox balance through endogenous enzymatic and non-enzymatic antioxidants. However, the increasing burden of oxidative stress caused by factors such as environmental pollutants, unhealthy diets, and chronic diseases, can overwhelm these systems. Under such conditions, exogenous antioxidants derived from diet or supplementation become important (20). Based on their origin, antioxidants are broadly classified as synthetic or natural. Synthetic antioxidants are chemically produced and are commonly used to prevent lipid oxidation, particularly in food systems. In contrast, natural antioxidants, derived from foods and medicinal plants, are regarded as bioactive compounds associated with metabolic benefits and a reduced risk of disease (22). Antioxidants counteract oxidation through two main mechanisms: by directly neutralizing free radicals (primary antioxidants) or via indirect pathways. Primary antioxidants act as radical scavengers and are consumed during the oxidation process. Secondary antioxidants operate through complementary mechanisms, including metal chelation, reactive species scavenging, hydroperoxide decomposition, UV absorption, and singlet oxygen quenching (23). However, the therapeutic efficacy of antioxidants remains inconsistent, as their effects are highly dependent on dosage, bioavailability, and disease context. These inconsistent outcomes likely reflect limited specificity and the complexity of ROS biology (22, 24).

## 3 N-acetylcysteine

N-acetylcysteine is a potent antioxidant that exerts cytoprotective effects by modulating redox homeostasis. It is a stable, synthetic derivative of L-cysteine, an endogenous amino acid essential for the synthesis of glutathione (GSH), one of the most important intracellular antioxidants. NAC primary activity is its antioxidant property. It indirectly increases GSH synthesis by providing cysteine as a precursor, and can also act as a direct scavenger of ROS and reactive nitrogen species, particularly when GSH levels are reduced. Additionally, NAC cleaves disulfide bonds in proteins, especially in mucin glycoproteins, thereby reducing mucus viscosity and exerting mucolytic effects (25, 26). Accordingly, it was approved by the FDA in 1963 as an inhaled mucolytic agent and was later introduced as an intravenous antidote for paracetamol poisoning (27, 28).

Beyond these well-established clinical indications, NAC exhibits a wide range of pleiotropic effects, including regulation of mitochondrial function, reduction of inflammation and oxidative stress, modulation of apoptosis, and antimicrobial activity through inhibition of biofilm formation in the lungs (29, 30). It also modulates glutamatergic neurotransmission, improves synaptic plasticity and cognitive function, and is therefore being investigated as a potential therapeutic option for various neurological and psychiatric disorders, including addiction, mood disorders, schizophrenia, autism, and neurodegenerative diseases (31). Additionally, NAC has demonstrated cardiovascular benefits by modulating nitric oxide metabolism, inhibiting angiotensin-converting enzyme, exerting antiplatelet effects, and reducing oxidative stress and inflammation (32). Given its broad mechanisms of action, NAC represents a promising therapeutic agent with potential applications in numerous pathological conditions associated with oxidative stress and impaired redox homeostasis.

## 4 Effects of the combined application of hyperbaric oxygen therapy and N-acetylcysteine

In all studies presented, HBOT was administered using clinically relevant protocols, with pressures ranging from 2 to 2.8 ATA and session durations of 60–120 min, applied as acute, single, or intermittent treatments (33–48). Most studies were conducted in various animal models and experimental settings (33–45), while others investigated the effects of combined NAC and HBOT administration *in vitro* using cell culture models (46, 47). Only one study evaluated these effects in humans (48). Studies that specifically examined the combined effects of HBOT and NAC as therapeutic modalities in animal models are summarized in Table 1 (33–42).

**TABLE 1:** Effects of combined HBOT and NAC administration in different experimental animal models.

References	Experimental protocol	Antioxidant and HBO protocol	Main findings
da Rocha et al. (33, 34)	Random pattern skin flaps in Wistar albino rats *(HBOT, NAC, NAC + HBOT groups)	NAC, 300 mg/kg i.p. and HBOT exposure at 2.4 ATA, 120 min for the consecutive 7 days. NAC was administered during the same experimental period as HBOT.	HBOT improved skin flap survival by reducing apoptosis and necrosis, whereas NAC alone was associated with less favorable outcomes. Notably, combined treatment did not enhance the beneficial effect observed with HBOT.
Zhang and Gould (35)	Ischemic wound model in Sprague - Dawley rats *(HBOT, NAC, NAC + HBOT groups)	NAC, 150 mg/kg i.p. and HBOT exposure at 2.4 ATA, 90 min for the consecutive 3–14 days. NAC was administered during the same experimental period as HBOT.	HBOT reduces oxidative stress and MMP overexpression in ischemic wounds via a redox-dependent ROS/MAPK mechanism, with maximal modulation observed around day 7. The combined HBOT and NAC treatment abolished the beneficial effects of HBOT.
Zhao et al. (36)	Traumatic spinal cord injury in Wistar rats *(HBOT, NAC, NAC + HBOT groups)	NAC, 150 mg/kg i.p. as a single dose and HBOT exposure at 2.4 ATA, 90 min for the consecutive 2 days. NAC was administered immediately after injury induction, while HBOT was applied 6 h later.	HBOT and NAC provided neuroprotection after spinal cord injury, with the combined therapy producing the most beneficial effects. The combination significantly reduced apoptosis and oxidative stress, enhanced endogenous antioxidant activity, suppressed pro-inflammatory cytokines (TNF-α, IL-1β), increased IL-10 expression, improved morphology, and resulted in superior functional recovery.
Onur et al. (37)	Acute pancreatitis in Wistar rats *(HBOT, NAC, NAC + HBOT groups)	NAC, 1000 mg/kg i.p. 1 and 25 h after induction and HBOT exposure at 2.5 ATA, 90 min, 6 and 18 h after induction.	HBOT and NAC attenuated biochemical, oxidative, and histopathological markers of severe acute pancreatitis. Combined treatment produced the most pronounced protective effects, showing reduction of oxidative stress parameters and the lowest pancreatic injury scores.
Taslipinar et al. (38)	Acetaminophen-induced liver injury in Sprague-Dawley rats *(NAC, NAC + HBOT groups)	NAC, 100 mg/kg i.p. once daily and HBOT exposure at 2.8 ATA, 90 min, 2 sessions per day, for the consecutive 5 days. NAC and HBOT were administered 24 h after injury induction. NAC was administered during the same experimental period as HBOT.	NAC treatment significantly attenuated biochemical and inflammatory indicators of liver injury. Importantly, the combination of NAC and HBOT produced more pronounced reductions in pro-inflammatory cytokines and histological damage, suggesting an enhanced hepatoprotective effect.
Cermik et al. (39)	Acetaminophen-induced nephrotoxicity in Sprague - Dawley rats *(NAC, NAC + HBOT groups)	NAC, 100 mg/kg i.p. once daily and HBOT exposure at 2.8 ATA, 90 min, 2 sessions per day, for the consecutive 5 days. NAC and HBOT were administered 24 h after injury induction. NAC was administered during the same experimental period as HBOT.	NAC treatment significantly attenuated biochemical markers of renal injury and reduced inflammatory mediators. Importantly, the combined NAC and HBOT produced more pronounced protective effects than NAC alone, resulting in the lowest creatinine, urea, cytokine, and neopterin levels, as well as improved renal histology.
Niu et al. (40)	LPS-induced fever in New Zealand white rabbits *(NAC, NAC + HBOT groups)	NAC, 5 mg/kg i.v. 1 h before induction and HBOT exposure at 2.5 ATA, for 60 min, 30 min after induction.	NAC and HBOT significantly reduced fever. However, combined treatment almost completely suppressed the fever response.
Toledo-Blas et al. (41)	Systemic loxoscelism in Wistar rats *(NAC, NAC + HBOT groups)	NAC, 200 mg/kg i.p. and HBOT exposure at 2 ATA, 60 min, for the consecutive 7 days. NAC and HBOT were administered 30 min after the inoculation. NAC was administered during the same experimental period as HBOT.	HBOT significantly improved coagulation parameters, enhanced antioxidant defenses, reduced oxidative damage, attenuated COX overexpression, and promoted more evident histopathological recovery, particularly in pulmonary tissue. NAC treatment also mitigated oxidative stress and inflammatory responses but was generally less effective than HBOT. Importantly, combined treatment resulted in complementary protective effects, showing improved oxidative balance, suppression of inflammatory markers, normalization of biochemical parameters, and the most notable structural recovery in lung tissue.
Balkan et al. (42)	Acute necrotizing pancreatitis in Sprague - Dawley rats *(NAC, NAC + HBOT groups)	NAC (dose not specified) and HBOT exposure at 2.5 ATA, 90 min, 2 sessions per day for the consecutive 5 days. NAC and HBOT were administered 6 h after injury induction. NAC was administered during the same experimental period as HBOT.	HBOT and NAC, or their combination significantly improved pulmonary antioxidant defenses and attenuated histological lung damage. However, differences were not observed between the single and combined treatment, indicating that the combination did not have additional protective effects.

*Each experimental design included appropriate control/model groups. NAC, N-acetylcysteine; HBOT, hyperbaric oxygen therapy; MMP, matrix metalloproteinase; ROS, reactive oxygen species; MAPK, mitogen-activated protein kinase; TNF-α, tumor necrosis factor-alpha; IL-1β, interleukin-1 beta; IL-10, interleukin-10; COX, cyclooxygenase.

Hyperbaric oxygen therapy and NAC interact through complex, redox-dependent mechanisms, and their combined effects are highly context-dependent. Across experimental studies, three general interaction patterns have been identified: attenuation of HBOT efficacy by NAC, beneficial combined effects under conditions of increased basal oxidative stress, and a pronounced dependence on both the timing and dosage of NAC administration relative to HBOT.

In several experimental models of tissue repair, NAC attenuated or abolished the beneficial effects of HBOT. Da Rocha et al. (33, 34) demonstrated, in a rat skin flap survival model with simultaneous administration of HBOT and NAC over 7 days, that HBOT significantly reduced apoptosis and necrosis, whereas the combination of NAC and HBOT did not improve outcomes. A similar effect was reported by Zhang and Gould (35) in a rat ischemic wound model, in which HBOT and NAC were administered concurrently for 14 days. HBOT markedly reduced oxidative stress in the ischemic wound, accompanied by decreased expression of several matrix metalloproteinases (MMP-1, MMP-2, MMP-8, and MMP-9) and increased expression of tissue inhibitor of metalloproteinases-2. This shift led to reduced extracellular matrix degradation and enhanced collagen formation in the wound. Notably, NAC did not enhance the effect of HBOT, rather, it attenuated HBOT-induced redox signaling. These findings suggest that ROS are important signaling molecules in tissue repair and that strong antioxidant intervention may disrupt redox-dependent mechanisms through which HBOT improves the healing of ischemic wounds. HBOT appears to improve perfusion and survival of ischemic skin flaps, most likely by increasing tissue oxygen partial pressure and enhancing oxygen diffusion through the interstitial space, while the initial pro-oxidant effect of HBOT may play an important role in triggering adaptive cellular responses (33–35).

Comparable findings have been reported in models of cerebral ischemia and skeletal muscle injury, where administration of NAC 30 min prior to HBOT suppressed ROS-dependent signaling pathways, including HIF-1α activation, angiogenesis, and tissue regeneration (43, 44). *In vitro* studies further support this concept. HBOT-induced ROS production enhances angiogenic potential and growth factor expression in mesenchymal stem cells, whereas NAC significantly diminishes these responses (46). Notably, a dose-dependent effect of NAC has been demonstrated in human dermal fibroblasts, where low concentrations of NAC combined with HBOT enhanced cell proliferation, while higher concentrations attenuated proliferation and reduced vascular endothelial growth factor production (47). Consistent with these findings, Thom et al. showed that NAC blunts the acute pro-oxidative signaling induced by hyperbaric oxygen exposure (45).

In contrast, under conditions marked by substantially elevated baseline oxidative stress, combined NAC and HBOT may provide additional therapeutic advantages (36–41). In a rat model of traumatic spinal cord injury, early administration of NAC and HBOT demonstrated the most pronounced protective effect. NAC was administered as a single dose immediately after injury, while HBOT was initiated 6 h later and continued for 2 days. Both interventions independently improved histological, biochemical, and functional parameters compared to untreated controls, but the combined treatment produced the most significant benefit, including reduced lipid peroxidation, enhanced antioxidant defense, decreased neuronal apoptosis, and a marked anti-inflammatory response (36). The observed effects in this study likely reflects differences in pathophysiological context and treatment timing relative to previous reports (33–35). In acute spinal cord injury, the initial phase is characterized by profound oxidative stress, inflammation, and mitochondrial dysfunction. In this context, NAC acts primarily as a potent antioxidant and mitochondrial stabilizer, while HBOT simultaneously increases tissue oxygen availability and activates reparative and neurotrophic mechanisms. Consequently, NAC in this model likely mitigates pathological lipid peroxidation and inflammation, rather than interfering with beneficial redox signaling, thereby causing HBOT to exert stronger neuroprotective and recovery-promoting effects (36). Comparable additive effects were reported by Onur et al. (37) in a rat model of acute necrotizing pancreatitis. When NAC and HBOT were administered during the early phase of injury within the first 24 h, both treatments separately reduced biochemical and histological markers of pancreatitis, while the combined therapy produced the most robust protective outcome. Similar protective effects were also observed in rat models of acetaminophen-induced acute liver injury (38) and nephrotoxicity (39). However, these studies (38, 39) did not include a group treated with HBOT alone, making it difficult to distinguish true synergistic interactions from additive effects. Further support for context-dependent benefit is provided by Niu et al. (40), who found that pretreatment with NAC prior to induction of fever and subsequent application of HBOT resulted in enhanced anti-inflammatory and antipyretic responses (40). Likewise, in a model of systemic loxoscelism, combined therapy attenuated oxidative stress and inflammatory reactions, although not all parameters demonstrated superiority over monotherapy (41). Balkan et al. (42) showed that NAC and HBOT improved antioxidant status and lung morphology in an experimental model of acute necrotizing pancreatitis, but did not confer a significant advantage over either therapy alone.

Importantly, clinical evidence remains extremely limited. The only available human study provides particularly valuable insight into the translational relevance of previously reported findings. In patients with type 2 diabetes and chronic non-healing foot ulcers, Efrati et al. (48) demonstrated that most patients showed a substantial increase in tissue oxygenation during HBOT. However, about one-third showed an insufficient response. This insufficient response was associated with increased oxidative stress, reduced total antioxidant capacity, and decreased nitric oxide bioavailability. In this subgroup, administration of NAC prior to and during HBOT significantly reduced oxidative stress, restored NO availability, and markedly improved tissue oxygenation. In contrast, patients who already demonstrated an adequate response to HBOT did not receive additional benefit from NAC administration, which directly supports the concept that NAC may be selectively advantageous in patients with pre-existing oxidative imbalance.

**FIGURE 2:**
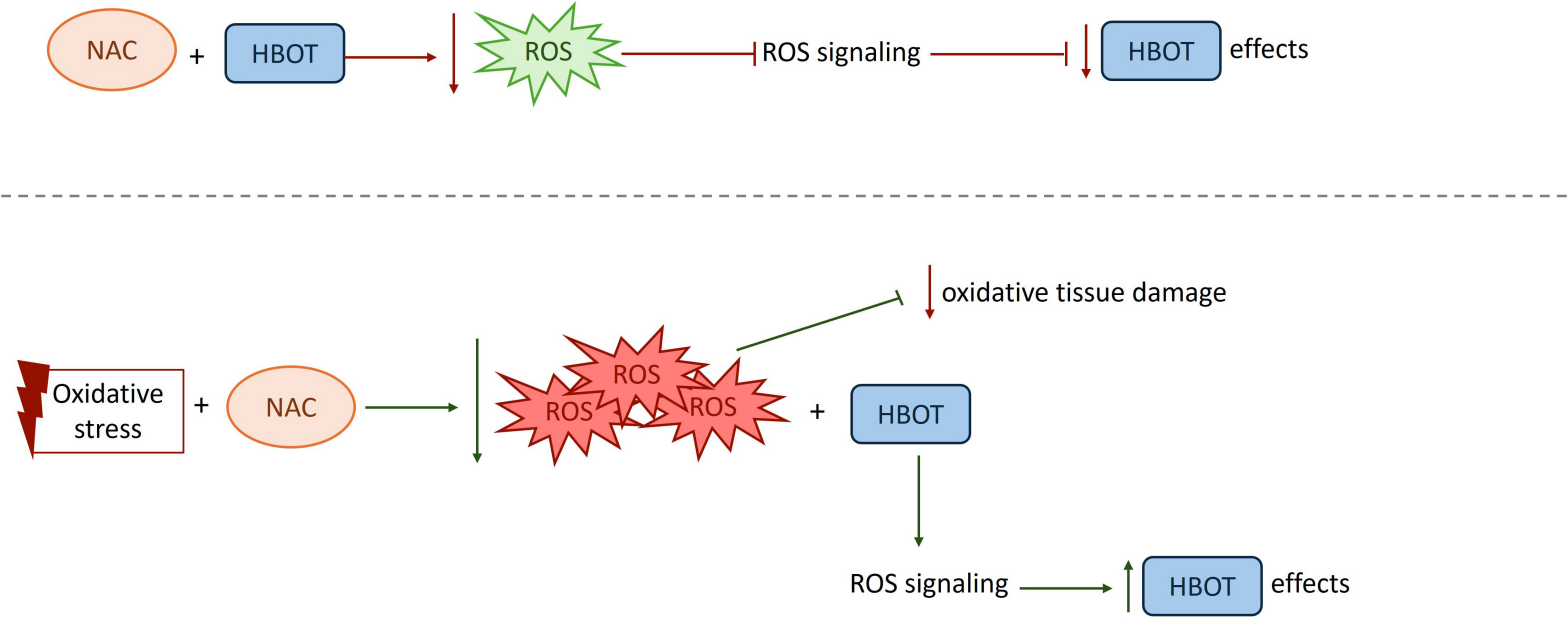
Context-dependent effects of combined N-acetylcysteine (NAC) and hyperbaric oxygen therapy (HBOT). The effects of NAC in combination with HBOT depend on baseline redox status, dose, and timing of administration. When given prior to HBOT, NAC may suppress the initial ROS burst required for adaptive signaling, thereby attenuating HBOT efficacy. In contrast, under conditions of elevated oxidative stress or when administered after injury, NAC can reduce excessive ROS, while preserving HBOT-induced signaling, resulting in adaptive and protective responses.

The interaction between HBOT and NAC can be conceptualized as a balance between the dual roles of ROS. HBOT-induced ROS generation may initiate adaptive signaling pathways that promote angiogenesis, tissue repair, and cellular survival, whereas excessive ROS accumulation contributes to oxidative damage and impaired microcirculation. NAC may enhance HBOT effects when oxidative stress is excessive and pathological, but may inhibit HBOT-induced benefits when administered in a manner that suppresses necessary redox signaling (Figure 2). These findings underscore the importance of baseline oxidative status, timing, and dose in determining the outcome of combined HBOT and NAC therapy.

## 5 Discussion

Overall, HBOT primarily serves as a redox-modulating stimulus. Acute exposure induces a transient pro-oxidant state, while repeated treatments are generally well tolerated, likely due to hormetic adaptation (4–13). This adaptation, though, is not universal and appears to depend on baseline inflammatory and oxidative status. In patients with severe inflammation or high baseline oxidative stress, compensatory mechanisms may be insufficient, resulting in persistent redox imbalance (18). For instance, Mrakic-Sposta et al. (18) showed that, in individuals with long COVID-19, repeated HBOT sessions reduced oxidative stress markers over time. However, this adaptive response was absent in a patient with severe osteomyelitis, suggesting that excessive inflammatory burden may hinder redox adaptation.

Optimization of HBOT treatment protocols, including pressure levels, exposure duration, and session frequency, represents a practical strategy for minimizing oxidative injury while preserving therapeutic efficacy (2). Furthermore, monitoring oxidative stress biomarkers during prolonged or intensive HBOT may help identify patients at increased risk and enable individualized treatment adjustments (19). Preclinical studies consistently demonstrate that HBOT combined with antioxidant interventions can synergistically mitigate oxidative stress, whereas clinical evidence remains limited and further trials are required to determine efficacy, optimal dosing, and translational relevance in humans. The findings presented in Section “4 Effects of the combined application of hyperbaric oxygen therapy and N-acetylcysteine” indicate that the interaction between HBOT and NAC is complex and is strongly influenced by baseline oxidative status, timing of administration, and dosing. Rather than representing a uniform pharmacological interaction, the combined effects of HBOT and NAC reflect a dynamic balance between two opposing roles of ROS. On one hand, ROS contribute to tissue injury under conditions of excessive oxidative stress, on the other, they function as essential signaling mediators that initiate adaptive and reparative pathways.

In experimental models characterized by high oxidative burden, such as systemic inflammation, toxic injury, and ischemia (36–41), NAC demonstrated a protective effect, and combined therapy was frequently associated with better outcomes than monotherapy. Similarly, in experimental models of acute kidney injury, chronic kidney disease, and traumatic spinal cord injury, other antioxidants such as apocynin, quercetin, and coenzyme Q10 have shown beneficial effects when administered concurrently with HBOT (49–53). In contrast, in studies examining tissue regeneration, angiogenesis, or HBOT preconditioning, ROS generated during hyperoxia appears to play a critical signaling role. Under these conditions, administration of NAC before or during HBOT may eliminate this redox signal, thereby weakening or completely abolishing the protective effects of HBOT (33–35, 43–46). This dichotomy highlights the importance of treatment timing, as early or concurrent antioxidant administration may interfere with adaptive signaling.

## 6 Conclusion

Overall, the analyzed literature suggests that the interaction between NAC and HBOT is neither universally synergistic nor antagonistic, but rather depends on the balance between pathological and signaling oxidative stress. In conditions where ROS is the dominant mechanism of tissue injury, antioxidant therapy may enhance the therapeutic response to HBOT. However, in models where ROS activates reparative pathways, excessive antioxidant modulation may weaken or block the adaptive effects of hyperbaric oxygenation. These findings highlight the importance of carefully considering dose, timing, and indication when combining antioxidants with HBOT. Given the relatively small number of studies conducted so far, further research is needed to clarify the specific interactions between HBOT and NAC in both experimental and clinical settings.
